# Nocturnal Heart Rate and Cardiac Repolarization in Lowlanders With Chronic Obstructive Pulmonary Disease at High Altitude: Data From a Randomized, Placebo-Controlled Trial of Nocturnal Oxygen Therapy

**DOI:** 10.3389/fmed.2021.557369

**Published:** 2021-03-01

**Authors:** Maya Bisang, Tsogyal D. Latshang, Sayaka S. Aeschbacher, Fabienne Huber, Deborah Flueck, Mona Lichtblau, Stefanie Ulrich, Elisabeth D. Hasler, Philipp M. Scheiwiller, Silvia Ulrich, Konrad E. Bloch, Michael Furian

**Affiliations:** Department of Respiratory Medicine, Sleep Disorders Center, University Hospital of Zurich, Zurich, Switzerland

**Keywords:** cardiac repolarisation, QTc prolongation, heart rate, hypoxia, chronic obstructive pulmonary disease

## Abstract

**Background:** Chronic obstructive pulmonary disease (COPD) is associated with cardiovascular disease. We investigated whether sleeping at altitude increases nocturnal heart rate (HR) and other markers of cardiovascular risk or arrhythmias in lowlanders with COPD and whether this can be prevented by nocturnal oxygen therapy (NOT).

**Methods:** Twenty-four COPD patients, with median age of 66 years and forced expiratory volume in 1 s (FEV_1_) 55% predicted, living <800 m underwent sleep studies at Zurich (490 m) and during 2 sojourns of 2 days each at St. Moritz (2,048 m) separated by 2-week washout at <800 m. During nights at 2,048 m, patients received either NOT (2,048 m NOT) or ambient air (2,048 m placebo) 3 L/min via nasal cannula according to a randomized, placebo-controlled crossover trial. Sleep studies comprised ECG and pulse oximetry to measure HR, rhythm, HR-adjusted QT interval (QTc), and mean oxygen saturation (SpO_2_).

**Results:** In the first nights at 490 m, 2,048 m placebo, and 2,048 m NOT, medians (quartiles) of SpO_2_ were 92% (90; 94), 86% (83; 89), and 97% (95; 98) and of HR were 73 (66; 82), 82 (71; 85), and 78 bpm (67; 74) (*P* < 0.05 all respective comparisons). QTc increased from 417 ms (404; 439) at 490 m to 426 ms (405; 440) at 2,048 m placebo (*P* < 0.05) and was 420 ms (405; 440) at 2,048 m NOT (*P* = NS vs. 2,048 m placebo). The number of extrabeats and complex arrhythmias was similar over all conditions.

**Conclusions:** While staying at 2,048 m, lowlanders with COPD experienced nocturnal hypoxemia in association with an increased HR and prolongation of the QTc interval. NOT significantly improved SpO_2_ and lowered HR, without changing QTc. Whether oxygen therapy would reduce HR and arrhythmia during longer altitude sojourns remains to be elucidated.

## Introduction

Altitude and air travel for professional and leisure activities is common, exposing millions of people to hypoxemia with potential adverse consequences on their health. Patients with pre-existing chronic obstructive pulmonary disease (COPD) may be at particular risk because of their impaired pulmonary gas exchange even at sea level. In patients with COPD, mortality increases almost linearly with an increasing resting heart rate (HR), with a hazard ratio of 1.39 (95% CI 1.21–1.60) above 80 bpm (1, 2). COPD patients also frequently reveal cardiac repolarization disturbances, including a prolonged QTc interval already near sea level, and this correlated inversely with arterial oxygen saturation (SpO_2_) (3). When exposed to a reduced barometric pressure at altitude or during air travel, patients with COPD are even more hypoxemic than healthy individuals due to impaired pulmonary gas exchange, in particular during sleep (4). Therefore, excessive hypobaric hypoxemia may put COPD patients at risk of an exaggerated HR increase (5) and possibly prolonged QTc and arrhythmias as seen in patients with obstructive sleep apnea (6), although this has not been specifically studied. In the presence of nocturnal hypoxemia and cardiac repolarization disturbances, nocturnal oxygen therapy (NOT) reversed nocturnal hypoxemia and reduced QTc prolongation in patients with pulmonary hypertension (7); however, whether NOT prevents hypoxemia-related cardiac repolarization disturbances in patients with COPD staying overnight at moderate altitude has never been studied.

Therefore, the current study evaluated the hypothesis that, compared with low altitude, sleeping at high altitude increases nocturnal HR and other markers of cardiovascular risks derived from the ECG including the QTc and arrhythmias in lowlanders with COPD and that these effects can be prevented by NOT during the stay at altitude.

## Methods

### Study Design

This study was part of a randomized, placebo-controlled, crossover trial evaluating the effect of altitude (vs. lowland) and NOT on nocturnal oxygenation and sleep-related breathing disorders in patients with COPD staying at 2,048 m for 2 consecutive days and nights (ClinicalTrials.gov NCT02150590) (8). The present study focuses on nocturnal HR, cardiac repolarization, and arrhythmia, which have not been addressed previously. The study was conducted from January 1 to October 31, 2014. Patients were studied in Zurich (490 m, baseline) and in St. Moritz (2,048 m) during 2 sojourns of 2 days/nights each while receiving either NOT or placebo (sham oxygen and ambient air) according to a randomized, crossover design with a 2-week washout period spent at <800 m between stays at 2,048 m. The sleep studies and daytime examinations took place in quiet and comfortable rooms at the University Hospital Zurich and in a mountain hostel in St. Moritz under convenient conditions (490 m, mean ± SD barometric pressure of 722 ± 2 mmHg, temperature of 24.9 ± 1.0°C, humidity 42 ± 5%; and 2,048 m, mean barometric pressure of 595 ± 3 mmHg, temperature of 25.5 ± 1.6°C, humidity of 35 ± 6%). Participants gave written informed consent, and the study was approved by the Cantonal Ethics Committee Zurich (EK 2013-0088).

### Interventions

During nights at 2,048 m, either NOT or ambient air (placebo) was administered at a flow rate of 3 L/min via nasal cannula. NOT or placebo was delivered via tubing by a concentrator located in a separate room out of the patient's sight (EverFlo, Philips Respironics, Zofingen, Switzerland). During the study, patients continued their usual medication.

For safety reasons, patients experiencing adverse effects at 2,048 m such as severe hypoxemia (SpO_2_ <75% for >30 min), acute mountain sickness (AMS) diagnosed by an AMSc score ≥0.7 (see below), high blood pressure (systolic >200 mmHg, diastolic >110 mmHg), or any other discomfort or intercurrent illness received oxygen and other therapy as appropriate and were evacuated to low altitude at the earliest convenience and withdrawn from the study.

### Participants

COPD patients, living at low altitude (<800 m), diagnosed according to the Global Initiative for Chronic Obstructive Pulmonary Disease (GOLD) criteria, grades 2–3, 18–75 years of age, both sexes, were recruited among outpatients of the Pulmonary Division, University Hospital Zurich, and surrounding hospitals.

Patients with COPD GOLD grade 4 or 1, severe gas exchange (SpO_2_ at 490 m <92% and/or PaCO_2_ >6 kPa at 490 m), more than mild or inadequately controlled cardiovascular disease, obstructive sleep apnea syndrome, current heavy smoking (>20 cigarettes per day), and previous intolerance to high altitude and stayed at altitude >1,500 m for >2 days within 4 weeks before the study or pregnancy were excluded from the study.

### Measurements and Outcomes

Clinical examinations and spirometry (9) (Blue Cherry, Geratherm Medical AG, Gschwenda, Germany) were performed. AMS was evaluated by the environmental symptoms cerebral score, which rates 11 questions on symptoms; a value of ≥0.7 in the weighted sum of responses reflects clinically relevant AMS (10). An arterial blood gas analysis was obtained (RapidPoint500, Siemens HealthCare, Zurich, Switzerland) in the morning after sleep studies at 490 m and at 2,048 m after NOT and placebo treatment.

During the first night at each location, polysomnography was performed including neurophysiologic standard derivations [2 central electroencephalogram (EEG) leads, electrooculogram (EOG), and electromyogram (EMG)], chest wall excursions by inductance plethysmography, and transcutaneous capnography (PtcCO_2_), pulse oximetry to measure SpO_2_ and record finger plethysmographic pulse waveforms (PWs) together with a 4-channel ECG (leads I–III, aVL, aVR, and aVF) recorded at 200-Hz sampling rate (Alice 5, Philips Respironics, Zofingen, Switzerland) (11). Mean nocturnal SpO_2_, the number of oxygen desaturation events per hour [the oxygen desaturation index (ODI), >3% dips], apnea/hypopnea, and sleep stages were scored according to international standards.

**Figure 1:**
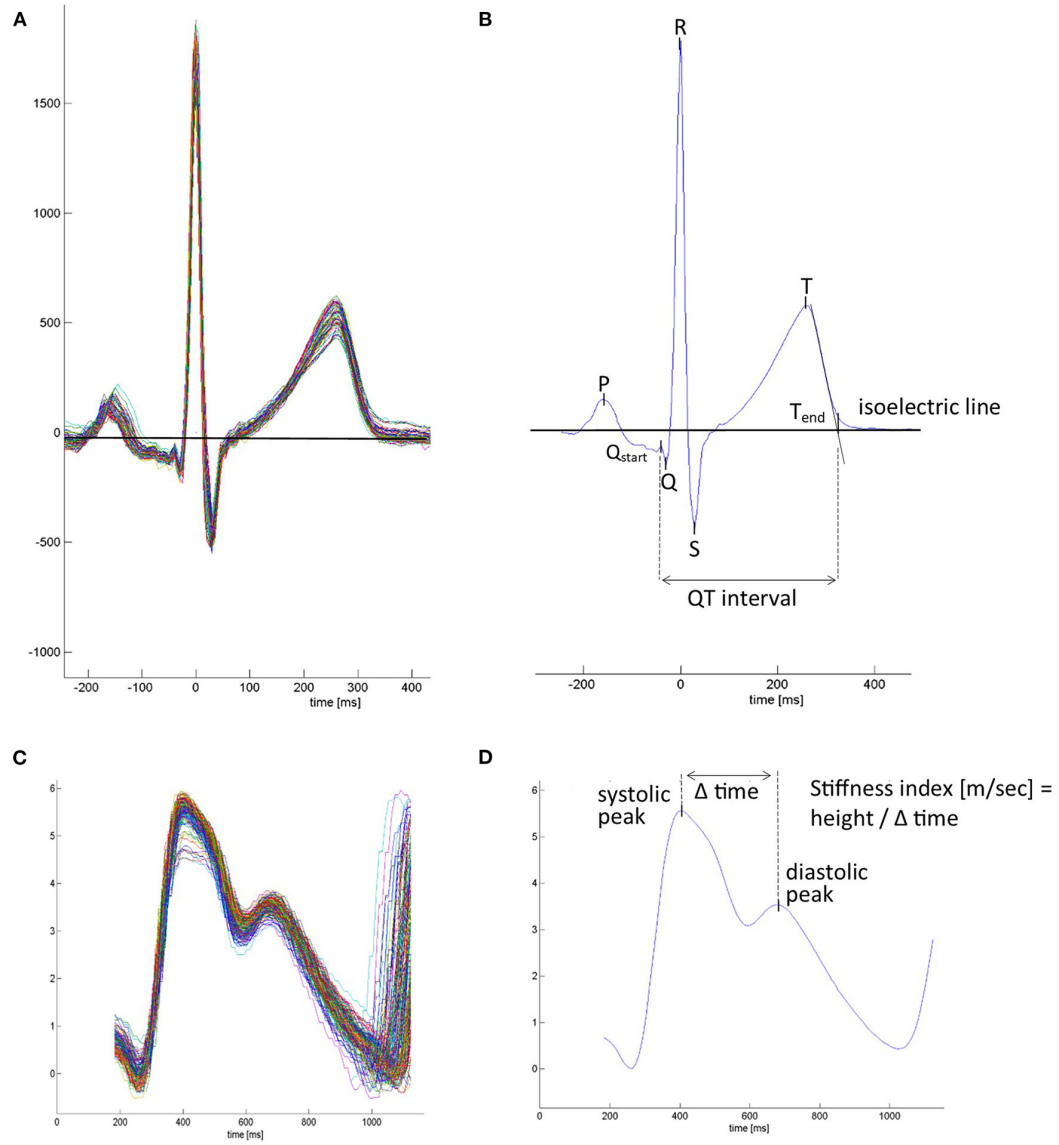
Mean waveforms from ECG-triggered ensemble averaged ECG and plethysmographic pulse wave. **(A)** Superimposed beat-by-beat ECG over a 1-min period. **(B)** Averaged mean ECG waveform over a 1-min interval. ECG markers were placed, and ECG-derived parameters were calculated. P, p-wave; Q_start_, the beginning of the Q wave and start of the QT interval; Q-R-S, QRS complex; T, t-wave peak; T_end_, end of t-wave and end of the QT interval, set at the connection point between the tangent of the steepest downslope of the T wave and the isoelectric line. **(C)** Superimposed beat-by-beat pulse waves. **(D)** Average pulse wave contour over a 1-min interval. Pulse wave-derived parameters were calculated. In case the diastolic maximum presented no peak, it was placed at the inflection point after the systolic peak.

Time series of overnight recordings of ECG and PW were processed by custom-built MATLAB program during the first night at 490 and 2,048 m under NOT or placebo. The software automatically detected the R wave of each QRS complex and ensemble averaged the ECG and corresponding PW cycles over successive 1-min intervals throughout the night. From ECG lead II (or lead I in case of artifacts), the QT interval was measured from the earliest onset of the Q wave until the intersection point between the isoelectric line and the tangent of the downslope of the T wave (Figure 1). HR-adjusted QT intervals (QTc), computed by Bazett's formula [QTc (ms) = QT (ms)/√RR interval (s)] (12), were determined minute by minute and overnight means, and 90th and 95th percentiles of 1-min means of QTc computed. In PW curves, the peak-to-peak time [PPT (ms), from systolic maximum to diastolic maximum or inflection point], the stiffness index [SI (m/s), body height divided by PPT] (13, 14), and the pulse transit time (PTT) measured from the ECG R-peak to the time at 50% PW amplitude were determined (Figure 1) and calculated. Arrhythmic events defined as supraventricular and ventricular extrasystoles (SVES and VES), and complex arrhythmias [CAs; bigeminy, trigeminy, couplet, and triplets and ventricular tachycardia (if any were recorded)] were manually scored. Blood pressure was measured in the evening and morning after 15 min, with the patient in an awake, relaxed, and supine position with a sphygmomanometer device.

The main outcomes of the current study were the QTc and other variables derived from the ECG and PW analyses, clinical evaluations, questionnaires, and results from sleep studies during the first night at each location. A separate sample size estimation was not performed for this study, as the number of required participants was determined for the main trial.

### Randomization and Blinding

Participants were randomized in balanced blocks of 4 by drawing an envelope containing 1 of 4 sequences of altitude exposure and treatment: (A) 490 m −2,048 m placebo−2,048 m NOT; (B) 490 m −2,048 m NOT−2,048 m placebo; (C) 2,048 m placebo−2,048 m NOT−490 m; and (D) 2,048 m NOT−2,048 m placebo−490 m. Patients and assessors were blinded to the treatment until conclusion of data analysis.

### Data Analysis and Statistics

Data were analyzed by the per-protocol approach including data from all participants completing the first night at 490 m and 2,048 m under NOT and placebo. Results are presented as median (quartiles). To evaluate overall effects between 490 m, 2,048 m placebo, and 2,048 m NOT, repeated-measures ANOVA was performed followed by paired *t*-test or Wilcoxon signed rank test. Multivariable regression analysis was used to evaluate the effects of altitude and NOT on outcomes while controlling for elapsed time and baseline variables. A probability of *P* < 0.05 was considered statistically significant.

## Results

**Figure 2:**
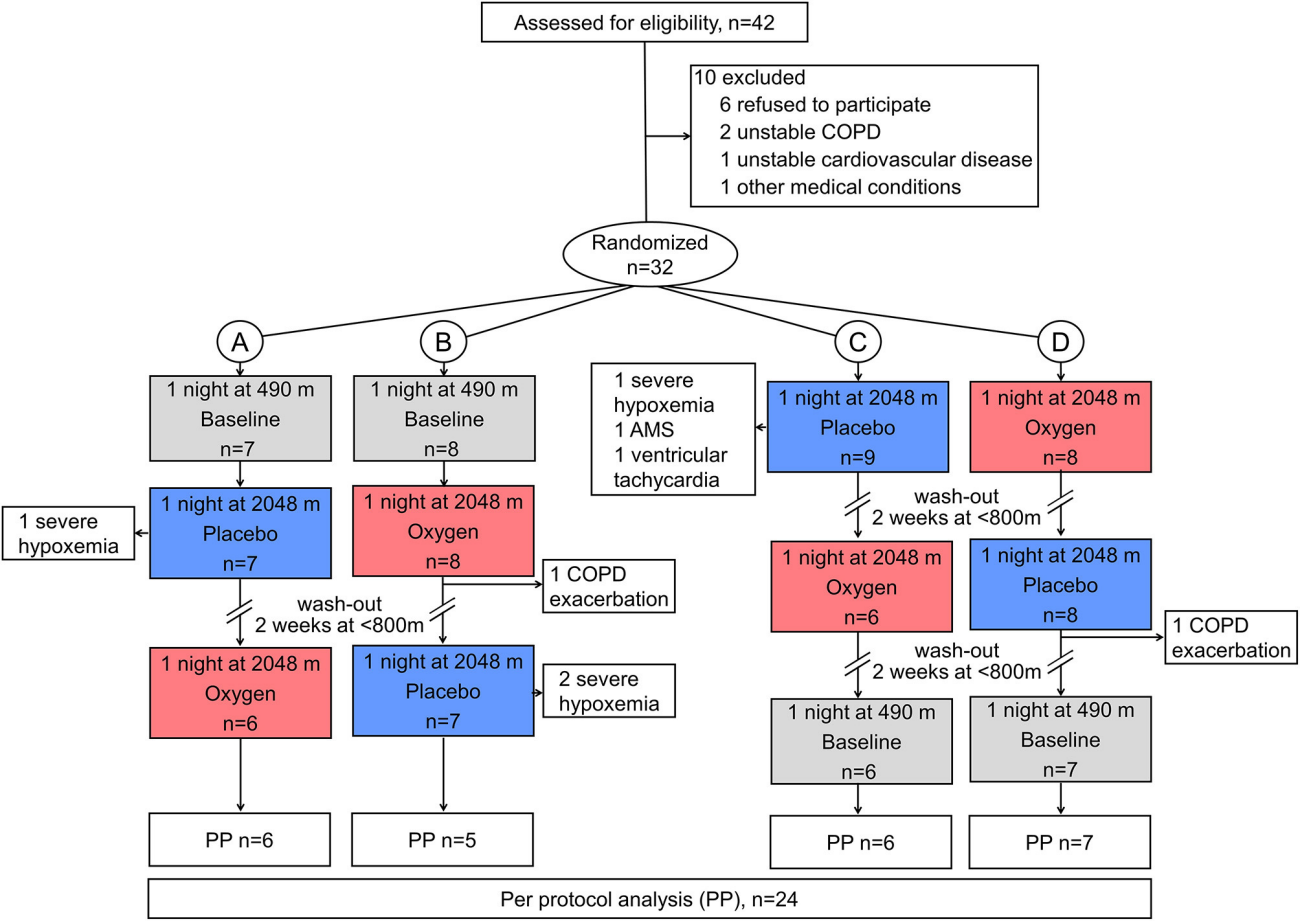
CONSORT flowchart. Altitude allocation sequence **(A–D)** and order of intervention (oxygen, red color; or placebo, blue color) at 2,048 m were randomized. After evaluations at 2,048 m, a 2-week washout period at <800 m was applied to avoid carryover effects. COPD, chronic obstructive pulmonary disease; PP, per protocol.

Forty-two patients were assessed for eligibility, and 10 were excluded for various reasons (Figure 2). At 2,048 m under placebo therapy, 8 patients had to be withdrawn from the study because of adverse events, including severe hypoxemia (*n* = 4, SpO_2_ <75% for >30 min), COPD exacerbation (*n* = 2), AMS (*n* = 1), and nocturnal non-sustained ventricular tachycardia (*n* = 1). The patient experiencing nocturnal ventricular tachycardia (mean nocturnal QTc prolongation of 448 ms) was referred to a cardiologist and started beta-blocker therapy. One additional patient completed all examinations and was included in the final analysis; however, he experienced a panic attack at 2,048 m after placebo. At 2,048 m under NOT, one patient experienced a COPD exacerbation. As previously published, NOT significantly reduced the proportion of altitude-related illnesses compared with placebo intervention (8 vs. 1, *P* < 0.001) (15). At 2,048 m, patients experiencing an altitude-related illness were treated with oxygen and medication as appropriate and were relocated to low altitude, which led to recovery without sequelae. Data of 24 patients, 11 men and 13 women, were analyzed (Figure 2). Participant characteristics are presented in Table 1.

**Table 1:** Patient characteristics.

**Variables**
*n*	24 (11 men, 13 women)
Age, years	66 (63; 70)
Body mass index, kg/m^2^	25.1 (21.4; 28.6)
FEV_1_, % predicted	55 (40; 62)
Smoking, pack-years	40 (26; 58)
Current smoker, *n* (%)	10 (42)
COPD GOLD, grade 2/grade 3, *n*	17/7
**Comorbidities**, ***n*** **(%)**	
Cardiovascular disease including hypertension	13 (54)
Diabetes	3 (13)
Depression	3 (13)
**Medication**, ***n*** **(%)**	
Inhaled glucocorticosteroids	2 (8)
Inhaled β-adrenergics	18 (75)
Inhaled anticholinergics	19 (79)
Diuretics	2 (8)
Antihypertensive medication	13 (54)
β-Blockers	1 (4)
Antidiabetics	3 (13)
Antidepressants	3 (13)

*Data presented as median (quartiles) and numbers (n)*.

*FEV_1_, forced expiratory volume in 1 s; COPD GOLD, Global Initiative for Chronic Obstructive Pulmonary Disease*.

**Figure 3:**
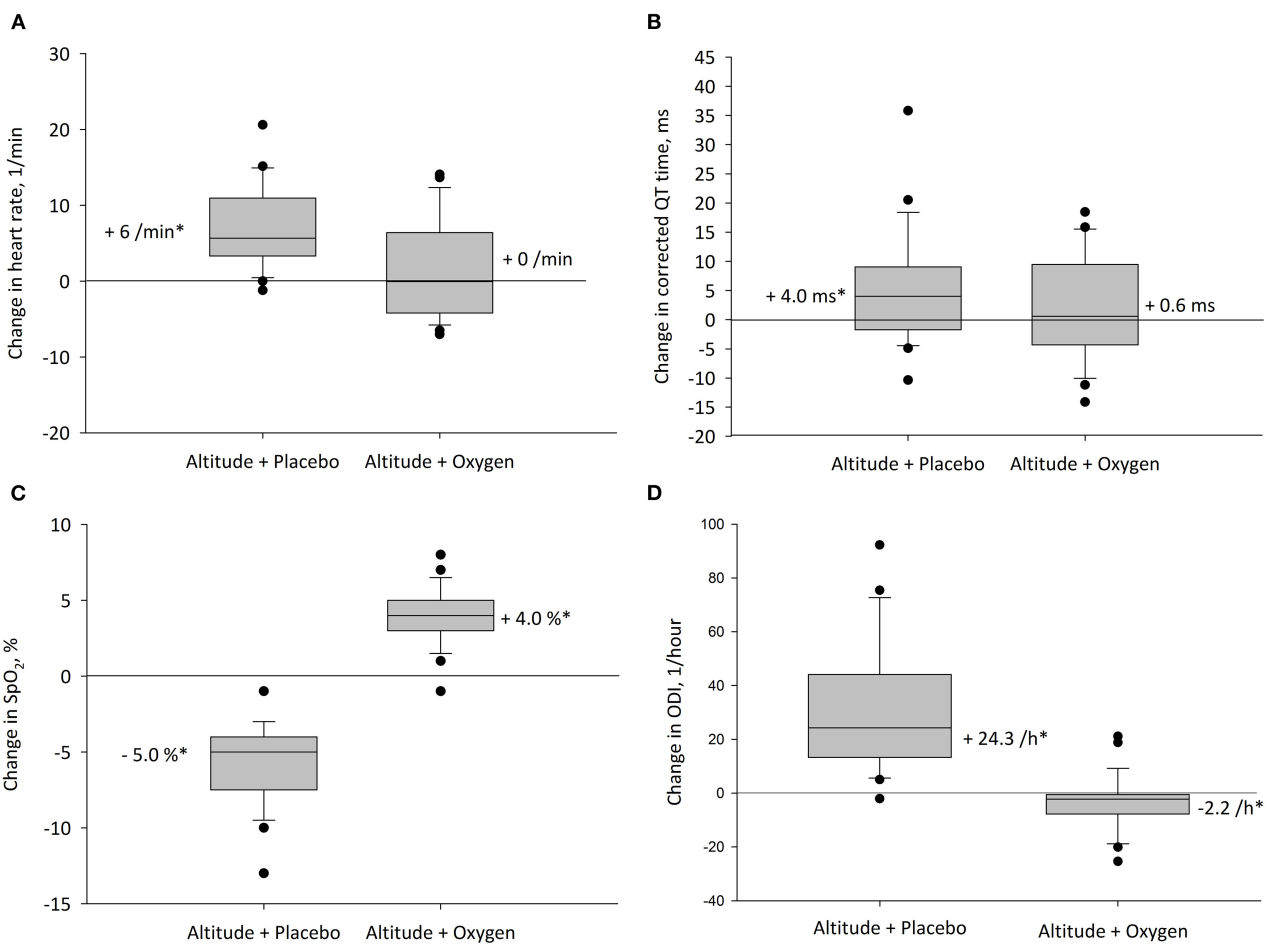
Altitude-induced changes in main outcomes. The box plots represent the changes between values at 490 and 2,048 m with corresponding treatment. Boxes and whiskers represent medians, quartiles, and 10th and 90th percentiles; dots represent individual values outside this range for **(A)** heart rate, **(B)** heart rate corrected QT interval (QTc), **(C)** oxygen saturation (SpO_2_), and **(D)** oxygen desaturation index (ODI). *P < 0.05 vs. 490 m.

**Figure 4:**
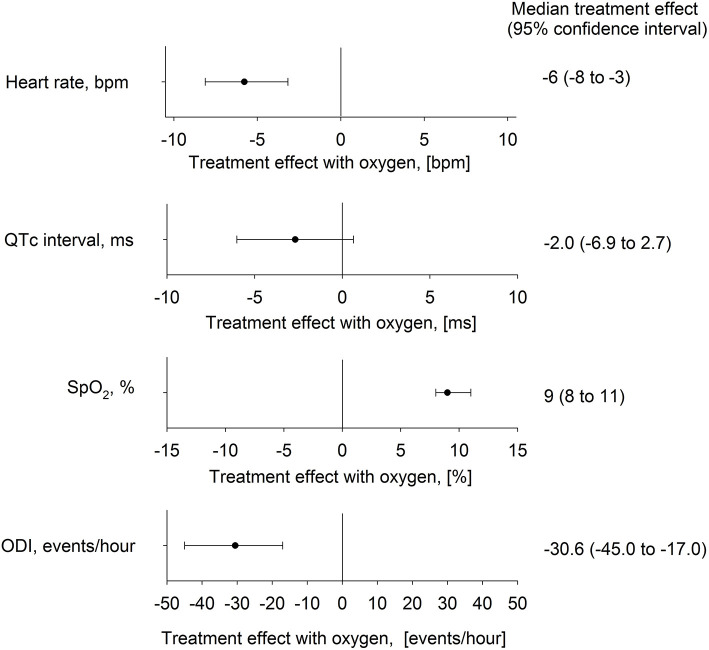
Effect of nocturnal oxygen therapy at 2,048 m on main outcomes. The plot shows median difference (95% confidence intervals) between altitude-induced changes during nights with oxygen and placebo for the heart rate, corrected QT interval (QTc), oxygen saturation (SpO_2_), and oxygen desaturation index (ODI).

The main results are summarized in Table 2 and illustrated in Figures 3, 4. With altitude exposure using placebo, there was a significant increase in nocturnal HR and prolongation of the mean, and the 90th and 95th percentiles of QTc. At 490 m and 2,048 m under placebo therapy, 1 of 24 (4%) patients had QTc prolongations exceeding the clinically relevant threshold of >450 ms in men or >460 ms in women compared with 0 of 24 patients while staying at 2,048 m under NOT (*P* = NS).

**Table 2:** Main outcomes derived from sleep studies during the first night and the following morning at each location.

**Variables**	**490 m**	**2,048 m placebo**	**2,048 m NOT**	***P*, ANOVA**
**ECG analysis**				
Heart rate, bpm	73 (66; 82)	82 (71; 85)^*^^¶^	78 (67; 84)	<0.001
QT duration, ms	380 (365; 410)	375 (355; 390)^*^^¶^	390 (370; 400)	<0.001
QTc duration, ms	417 (404; 439)	426 (405; 440)^*^	420 (405; 440)	0.028
90th percentile QTc, ms	422 (410; 436)	430 (406; 445)^*^	427 (405; 446)	0.016
95th percentile QTc, ms	424 (412; 438)	432 (408; 447)^*^	430 (408; 447)	0.024
QTc max, ms	439 (425; 455)	444 (418; 457)	442 (420; 452)	0.397
TpTe, ms	60 (55; 70)	60 (55; 75)	65 (60; 70)	0.581
TpTe c, ms	65 (62; 73)	68 (60; 79)	71 (61; 80)	0.836
VES, 1/h	0.3 (0.0; 2.4)	1.6 (0.1; 14.5)	0.3 (0.1; 2.9)	0.341
SVES, 1/h	1.8 (0.4; 10.0)	2.4 (0.4; 7.8)	2.2 (0.5; 7.5)	0.362
Complex arrhythmia, 1/h	0 (0; 0)	0 (0; 0)	0 (0; 0)	0.249
**Pulse wave analysis**				
Peak-to-peak time, ms	160 (150; 180)	160 (153; 178)	170 (160; 190)	0.559
Stiffness index, m/s	10.2 (9.2; 11.1)	10.5 (9.2; 11.1)	10.1 (8.9; 10.5)	0.388
Pulse transit time, ms	345 (337; 364)	332 (325; 351)^*^^¶^	352 (338; 365)	<0.001
Reflection index	0.74 (0.68; 0.79)	0.75 (0.62; 0.76)^*^	0.75 (0.65; 0.78)	0.034
**Nocturnal oxygenation and capnography**				
Mean nocturnal SpO_2_, %	92 (90; 94)	86 (83; 89)^*^^¶^	97 (95; 98)^*^	<0.001
ODI, 1/h	3.6 (1.3; 13.7)	37.8 (16.7; 59.5)^*^^¶^	0.6 (0.1; 2.6)^*^	<0.001
PtcCO_2_, mmHg	41 (37; 46)	44 (42; 48)^*^^¶^	48 (42; 52)^*^	<0.001
**Morning evaluation**				
BP systolic, mmHg	122 (111; 135)	125 (112; 137)	125 (116; 134)	0.679
BP diastolic, mmHg	70 (65; 77)	69 (62; 82)	69 (65; 80)	0.762
BP mean, mmHg	85 (80; 94)	86 (78; 98)	86 (82; 98)	0.695
**Arterial blood gases**				
pH	7.44 (7.43; 7.46)	7.47 (7.45; 7.49)^*^	7.47 (7.45; 7.48)^*^	<0.001
PaCO_2_, kPa	5.1 (4.7; 5.2)	4.7 (4.2; 7.9)^*^	4.5 (4.0; 5.0)^*^	<0.001
PaO_2_, kPa	9.0 (8.3; 9.8)	8.1 (7.3; 8.6)^*^	8.0 (7.2; 8.4)^*^	<0.001
SaO_2_, %	94.4 (92.5; 95.3)	90.1 (87.7; 92.1)^*^	90.6 (87.6; 91.8)^*^	<0.001

*Median (quartiles). Arterial blood gases were performed in the morning, 10 min after awakening and while breathing ambient air. 90th and 95th percentiles of QTc were calculated from the average 1-min QTc values over the complete night*.

*NOT, nocturnal oxygen therapy; QTc, corrected QT time using the Bazett formula; QTc max, the 1-min interval with the longest QT interval during the night; TpTe, time between Tpeak and Tend; TpTe c, TpTe corrected for heart rate using the Bazett formula; VES and SVES, ventricular and supraventricular arrhythmia; Complex arrhythmias, including bigeminy, trigeminy, couplets, and triplets; Peak-to-peak time, the time difference between the pulse wave contour-derived systolic and diastolic maximum; Stiffness index, calculated from the height of a participant divided by the Peak-to-peak time; Pulse transit time, the time between the ECG R-peak to the time at 50% pulse wave amplitude; Reflection index, calculated by dividing the diastolic amplitude by the systolic amplitude of the systolic and diastolic maximum derived from the pulse wave contour; SpO_2_, arterial oxygenation measured by finger oximetry; ODI, oxygen desaturation index defined as number of >3% dips in SpO_2_; PtcCO_2_, transcutaneous carbon dioxide; BP, blood pressure*.

* *P <0.05 vs. 490 m*;

¶ *P <0.05 vs. NOT*.

The mean nocturnal SpO_2_ was significantly lower at 2,048 m placebo compared with 490 m and 2,048 m NOT (*P* < 0.05 both comparisons), and SpO_2_ at 2,048 m NOT was higher compared with 490 m (*P* < 0.05, Figure 3C). The ODI was 3.6/h (1.3; 13.7) at 490 m, 37.8/h (16.7; 59.5) at 2,048 m placebo, and 0.6/h (0.1; 2.6) at 2,048 m NOT (*P* < 0.05 all comparisons, Figure 3D). The mean nocturnal HR was higher at 2,048 m placebo compared with 490 m and 2,048 m NOT (*P* < 0.05 both comparisons, Figure 3A). The PTT was reduced at 2,048 m placebo compared with 490 m and 2,048 m NOT (*P* < 0.05 both comparisons), and SI remained unchanged at 490 m, 2,048 m placebo, and 2,048 m NOT. QTc was prolonged at 2,048 m placebo compared with 490 m (Table 2, Figure 3B). NOT significantly reduced nocturnal HR, lowered the QT but did not modify the QTc interval, lowered ODI, and increased SpO_2_ at 2,048 m compared with placebo (Figure 4). No change in the number of arrhythmic events (VES, SVES, and CA) was observed at altitude with placebo or NOT compared with 490 m (Table 2).

**Figure 5:**
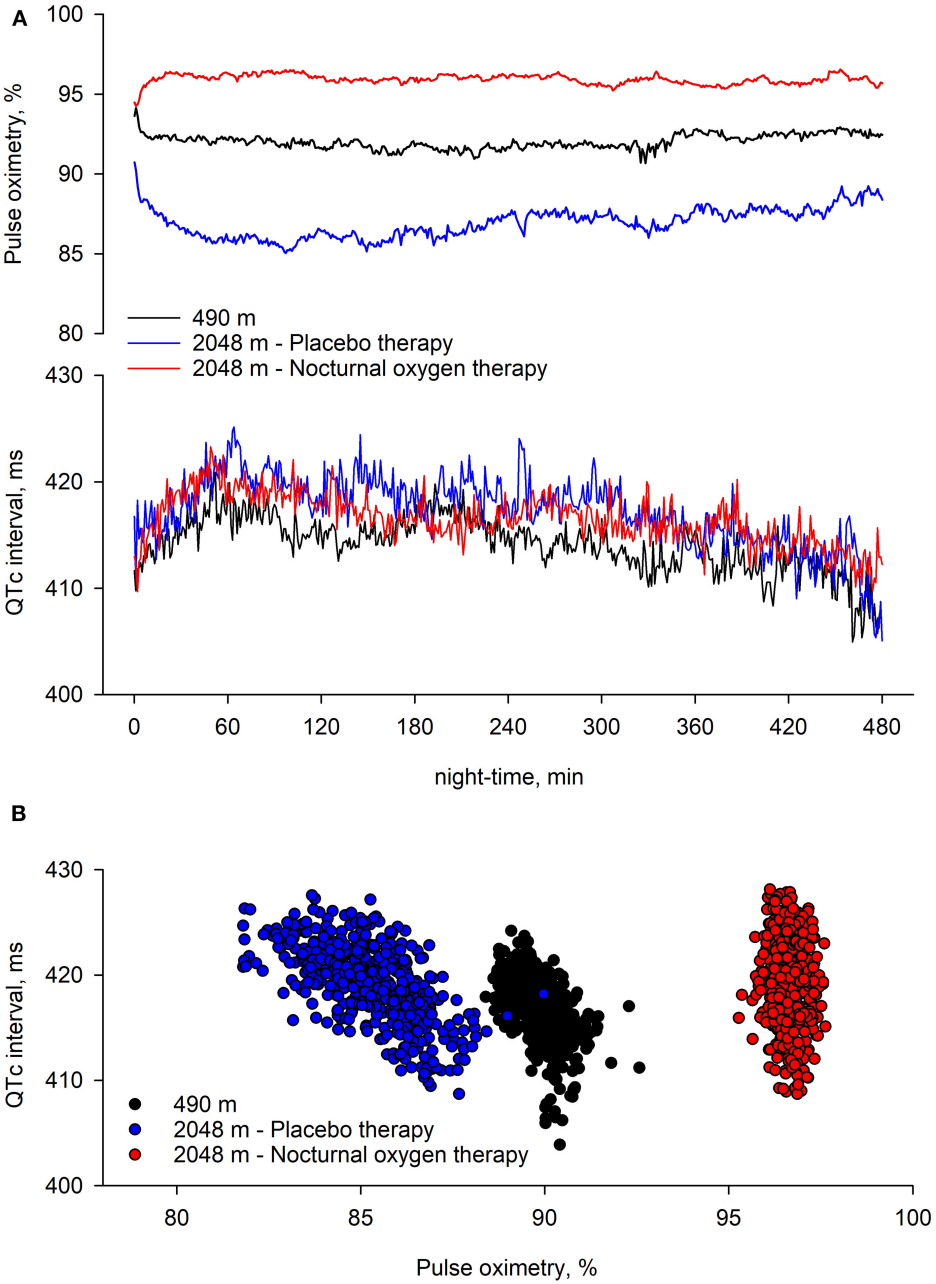
Nocturnal pulse oximetry and corrected QT interval in relation to the underlying intervention. Time series of group means of pulse oximetry and corrected QT interval (QTc) over consecutive 1-min periods during study nights are displayed. At 490 m, arterial oxygen saturation shortly decreases after lights off and remained lower until the end of the night. At 2,048 m under placebo therapy, arterial oxygen saturation shortly decreases after lights off and steadily improves over the night. At 2,048 m under nocturnal oxygen therapy, arterial oxygen saturation increases and remains higher until the end of the night. Compared with 490 m, at 2,048 m under placebo therapy, QTc remains elevated throughout the night; however, independent of the intervention, QTc increases in the 1 h of the night, while it progressively returns to lower values thereafter.

Multivariable regression analysis of consecutive 1-min means of QTc revealed that altitude was an independent predictor of an increased QTc; furthermore, it revealed that QTc increased in the early night and reversed to awake QTc values in the early morning hours of the night, independent of altitude or treatment and when controlled for age, sex, and forced expiratory volume in 1 s (FEV_1_) % predicted (Table 3, Figure 5).

**Table 3:** Night-time progression of QTc: mixed, linear regression analysis.

**Dependent variable: QTc, ms**	**Coefficient**	**SE**	**95% CI**	***P***
490 m (ref)				
2,048 m placebo vs. 490 m	4.7	0.1	4.5 to 4.9	<0.001
2,048 m NOT vs. 490 m	1.8	0.1	1.6 to 2.0	<0.001
Night-time				
First 10 min (ref)				
11–70 min	3.6	0.3	3.0 to 4.2	<0.001
71–130 min	4.0	0.3	3.4 to 4.6	<0.001
131–190 min	3.3	0.3	2.6 to 3.9	<0.001
191–250 min	3.2	0.3	2.6 to 3.8	<0.001
251–310 min	2.8	0.3	2.2 to 3.4	<0.001
311–370 min	0.9	0.3	0.3 to 1.5	0.003
371–430 min	0.1	0.3	−0.5 to 0.7	0.739
431–480 min	−1.0	0.3	−1.6 to −0.4	0.002
Age, years	0.9	1.4	−1.8 to 3.6	0.504
Sex, male vs. female	−17.2	15.5	−47.6 to 13.2	0.267
FEV_1_ %pred. at 490 m	−0.3	0.6	−1.5 to 0.9	0.664
Intercept	374.4	110.3	158.2 to 590.5	0.001

*The dependent variable QTc was averaged over consecutive 1-min intervals throughout the nights, and a total of 31,589 1-min intervals were included in this mixed, linear regression analysis*.

*FEV_1_, forced expiratory volume in the first second of expiration*.

## Discussion

This randomized, placebo-controlled crossover trial in patients with moderate to severe COPD demonstrated that sleeping at 2,048 m, an altitude of many tourist destinations and permanent settlements, is associated with hypoxemia, an increased nocturnal HR, a reduced PTT, and a moderate prolongation of the QTc interval. Reassuringly, a QTc prolongation above the limit considered clinically relevant was observed in only one patient (already at low altitude), and symptomatic arrhythmias were recorded in only 1 other patient. However, 8 (26%) of the COPD patients suffered from altitude-related illnesses requiring medical intervention and descent to lower altitude. NOT significantly reduced the incidence of altitude-related illnesses at 2,048 m compared with placebo, improved the SpO_2_, mitigated the altitude-induced increase in HR, and prevented the reduction in PTT but did not change the QTc.

A higher resting HR is a strong and independent predictor of mortality in patients with COPD with the relative risk increasing by 21% by every 10 bpm (1). A HR >80 bpm was associated with a significantly increased all-cause mortality compared with <80 bpm (hazard ratio 1.6, 95% CI 1.1–2.3). Comparably, in a large cohort of COPD patients at cardiac risk, resting HR above 80 bpm revealed a hazard ratio of 1.39 (1.21–1.60) (2). In the present study, we found a significant increase in mean nocturnal HR of, on average, 6 bpm when COPD patients traveled from 490 to 2,048 m. The resulting resting HR was 82 bpm, a level that may well be associated with adverse health events in the long run (1). Prospective long-term trials in patients with COPD staying at high altitude are required to elucidate whether the observed altitude-related HR increase represents a similar hazard as if it was developing at low altitude or whether it is a beneficial adaptation to hypoxia. The HR increase with altitude in the present COPD cohort was doubled compared with healthy exposed to even slightly higher altitudes of 2,590 m (16), and the findings confirm the exaggerated HR increase in COPD patients exposed to isocapnic hypoxia (5). When the present COPD patients collective used NOT at altitude, the mean nocturnal HR increase could be completely reversed to low-altitude levels <80 bpm, a reduction that can be considered relevant to decreased cardiovascular risk in the long run (1, 2). Consistent with an increased sympathetic excitation, the PTT was reduced at 2,048 m under placebo but not under NOT therapy.

In the general population, a prolonged QTc has been found to be a risk factor for ventricular fibrillation and sudden cardiac death (17, 18), although arrhythmias are more often associated with an even longer QTc of >500 ms (17). A clinically important threshold for QTc prolongation has been suggested to be >450 ms in men and >460 ms in women (19, 20). The values observed in the current COPD patients were well below this threshold at low and high altitudes; however, at moderate altitude, QTc might have been prolonged due to altitude-induced hypoxemia and its consequences on the cardiovascular and ventilatory system and possibly other, unknown factors.

It has been shown that COPD is associated with cardiac arrhythmias and specific ECG alterations that have prognostic significance (21). The prevalence of QTc prolongation in COPD patients in comparison with the general population (22) remains controversial since it has been suggested to be increased (3), reduced (23), or similar (24). In the currently studied cohort of mildly hypoxemic and non-hypercapnic patients with COPD, a low prevalence of clinically important prolongations in QTc was observed at low altitude. The mean QTc of the night exceeded the upper limit of normal in no patient, and the 95th percentile of nocturnal QTc exceeded the upper limit of normal in 2 of 24 patients. In a prospective 5-year cohort study including 246 patients with COPD, prolonged QTc and other QT-derived parameters were found to be predictors of poor survival (25). The association with prolonged cardiac repolarization and sudden cardiac death in COPD patients is however not yet confirmed and needs further investigation (3). Tirlapur and Mir (26) recorded nocturnal electrocardiographic changes in 12 severely hypoxemic and hypercapnic COPD patients at low altitude. In several of them, major QTc prolongations were noted, and these were improved by supplemental oxygen administration. Consistent with these observations, we found that aggravation of hypoxemia during exposure to hypobaric hypoxia at high altitude was associated with a QTc prolongation in the studied patients with COPD, which were mildly hypoxemic and non-hypercapnic. We can therefore confirm that altitude induced nocturnal hypoxemia and prolonged QTc; however, along with a significant HR reduction, we found no modification in the already relatively low QTc under NOT (27).

A previous study in COPD patients (28) and a recent meta-analysis (29) suggest that prolongation in the Tpeak to Tend intervals may predict sudden cardiac death. In the current study, Tpeak to Tend did not change with altitude exposure possibly because of a reduced sensitivity of this variable to hypoxemia.

COPD has been associated with a higher prevalence of cardiac arrhythmias, angina, acute myocardial infarction, congestive heart failure, stroke, pulmonary embolism, and an increased risk of death due to cardiovascular disease (30). In the current study, we found numerous short, self-limited arrhythmic events, mainly pre-mature beats, which were not altered by sleeping at high altitude, and thus our findings did not confirm some previous studies (26, 31, 32). We found no relevant sustained arrhythmias at both altitude with and without NOT; however, one patient had three runs of ventricular tachycardia during the night at 2,048 m with placebo therapy. This patient was referred to a cardiologist and commenced beta-blocker therapy.

The literature on ECG recordings during real or simulated altitude is scarce. In 8 COPD patients investigated at 1,920 m, no cardiac irregularity during daytime ECG recordings was observed (33). In studies evaluating the exposure of COPD patients to aircraft cabin conditions at 2,438 m altitude equivalent, no change in arrhythmias or ischemia signs (34) except for isolated SVES and VES (35) was found. However, no further information about the ECG characteristics at baseline was described. Gong et al. (36) studied the respiratory and cardiac response to altitude in 22 COPD patients. By giving the participants hypoxic gas mixtures corresponding to 1,524, 2,438, and 3,048 m, 10 out of 22 (45.5%) COPD patients developed asymptomatic cardiac arrhythmias, consisting of SVES and VES. Oxygen administration improved almost all physiologic indexes. In the present study, we found no relevant sustained arrhythmias at both altitudes with and without NOT. As QTc prolongation persisted despite the correction of nocturnal hypoxemia by NOT, other factors than hypoxemia associated with the stay at high altitude including alterations in intravascular volume, pH, and serum electrolyte concentrations (calcium and potassium) and further, unknown causes might have affected cardiac repolarization (37, 38).

Furthermore, we performed pulse wave analysis and assessed the arterial stiffness index SI, a marker previously shown to be associated with cardiovascular risk (14). Thus, the study by Clarenbach et al. showed a SI of 9.4 m/s in patients with 2 or more cardiovascular risk factors compared with 6.4 m/s in a control group without any cardiovascular risk factors. Our results confirm that COPD patients have an elevated SI of 10.2 m/s already at 490 m; however, moderate altitude and NOT intervention did not further change the SI, suggesting a low sensitivity to acute hypoxemia.

The limitations of our study are the relatively small sample size and the short exposure time to altitude. However, the randomized, crossover design allowed to reduce sample size, as each patient served as his/her own control. This study applied 3 L/min of NOT through a nasal cannula to improve hypoxemia and sleep-disturbed breathing despite possible mouth breathing. This intervention improved SpO_2_ and ODI (Table 2) beyond the values obtained at 490 m, suggesting that an even lower dose of NOT might be sufficient during altitude travel in certain patients. Investigating cardiac rhythms and repolarization during longer altitude stays would be interesting but was logistically not feasible. Patients with more severe COPD, exacerbation, or severe daytime hypoxia were not included in this trial, and thus further studies at higher altitudes and in patients with more severe COPD are necessary to widen the knowledge of effects of sleeping at altitude in more severe COPD on HR and indices of repolarization.

## Conclusions

This randomized, placebo-controlled crossover trial on the effect of a stay at altitude (2,048 m) in COPD patients on cardiac rhythm and repolarization revealed a significant increase in mean nocturnal HR and a prolongation of QTc within the normal range. Cardiac arrhythmias were generally uncommon and clinically relevant, requiring descent to lower altitude in only 1 of the 24 patients. Nevertheless, several patients experienced other altitude-related adverse effects on their health that were effectively prevented by NOT. Therefore, patients with moderate to severe COPD might consider using NOT during altitude sojourns.

## Data Availability Statement

The datasets presented in this article are not readily available because the raw data supporting the conclusions of this article include sensitive patient data. Requests to access the datasets should be directed to michael.furian@usz.ch.

## Ethics Statement

This study involved human participants and was reviewed and approved by Cantonal Ethics Committee Zurich (EK-2013-0088). The patients/participants provided their written informed consent to participate in this study.

## Author Contributions

MB and MF had full access to all the data in the study and take responsibility for the integrity of the data and the accuracy of the data analysis. KB, TL, SU, and MF: concept and design. All authors: acquisition, analysis, or interpretation of data, and critical revision of the manuscript for important intellectual content. MB, KB, and MF: drafting of the manuscript and statistical analysis. KB: obtained funding. KB, TL, SA, FH, DF, SU, and MF: administrative, technical, or material support and supervision.

## Conflict of Interest

The authors declare that the research was conducted in the absence of any commercial or financial relationships that could be construed as a potential conflict of interest.
